# Venturi Injector Optimization for Precise Powder Transport for Directed Energy Deposition Manufacturing Using the Discrete Element Method and Genetic Algorithms

**DOI:** 10.3390/ma17040911

**Published:** 2024-02-16

**Authors:** Joshua García-Montagut, Rubén Paz, Mario Monzón, Begoña González

**Affiliations:** 1 Department of Mechanical Engineering, University of Las Palmas de Gran Canaria (ULPGC), 35015 Las Palmas de Gran Canaria, Spain; mario.monzon@ulpgc.es; 2 SIANI University Institute, University of Las Palmas de Gran Canaria (ULPGC), 35015 Las Palmas de Gran Canaria, Spain; bego.landin@ulpgc.es

**Keywords:** additive manufacturing, directed energy deposition, venturi, discrete elements modeling, genetic algorithms, powder transport

## Abstract

Additive manufacturing technologies such as directed energy deposition use powder as their raw material, and it must be deposited in a precise and controlled manner. Venturi injectors could be a solution for the highly precise transport of particulate material. They have been studied from different perspectives, but they are always under high-pressure conditions and mostly fed by gravity. In the present study, an optimization of the different dimensional parameters needed for the manufacturing of a Venturi injector in relation to a particle has been carried out to maximize the amount of powder capable of being sucked and transported for a specific flow in a low-pressure system with high precision in transport. For this optimization, simulations of Venturi usage were performed using the discrete element method, generating different variations proposed by a genetic algorithm based on a preliminary design of experiments. Statistical analysis was also performed to determine the most influential design variables on the objective, with these being the suction diameter (D3), the throat diameter (d2), and the nozzle diameter (d1). The optimal dimensional relationships were as follows: a D3 34 times the particle diameter, a d2 26.5 times the particle diameter, a d1 40% the d2, a contraction angle alpha of 18.73°, and an expansion angle beta of 8.28°. With these proportions, an 85% improvement in powder suction compared to the initial attempts was achieved, with a maximum 2% loss of load.

## 1. Introduction

Additive manufacturing has been considered an important and growing form of manufacturing for many years. The standard ISO/ASTM 52900:2021 [1] defines it as the process by which, starting from the data of a 3D model, a part is manufactured layer by layer. However, the way to comply with this definition is extremely varied and depends on different technologies. This standard divides these technologies into seven categories, but only four of them can directly use their raw material in powder form: binder jetting, directed energy deposition, material extrusion, and powder bed fusion.

The materials used in these technologies can be metallic, polymeric, or ceramic, but all have in common their high quality and consequent cost.

**Figure 1:**
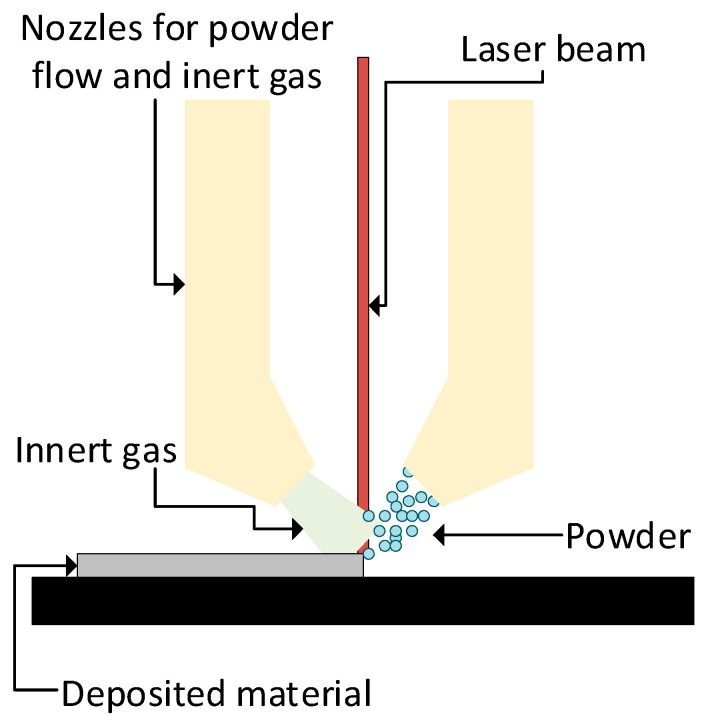
A schematic diagram of a DED system with a laser and powder feedstock.

Taking directed energy deposition as an example, this technology uses a combination of inner gas (normally argon) as a clean atmosphere to avoid oxidation, feeding material (metallic or polymeric powder [2]), and a power source to melt the powder in a controlled manner (a laser or electron beam). A schematic of a DED powder system is shown in Figure 1.

This technology divides the powder feed into three moments [3]: metering, conveyance, and delivery. Each moment has different possible methods (mechanical, pneumatic, vibration-assisted, or gravitational methods, for example), all of them with different control necessities. However, a high accurate spray control is needed because part of the sprayed powder is not used [4]. This manufacturing system could have multiple nozzles, being able to mix different materials to obtain Functionally Graded Materials (FGMs) [5,6].

Analyzing the three moments in the powder feed, a novel solution could be the use of Venturi injectors, which allow the powder to be sucked from one point and pushed to where it is needed in a controlled manner, unifying the metering and conveyance systems.

From analyzing the solutions on the market that use these technologies, it can be seen that the raw materials in powder format tend to have very small particle sizes and are available in all densities. These characteristics can greatly influence the design of Venturi injectors and must therefore be taken into account when sizing them.

The most studied point in the literature is the net efficiency of the Venturi injector, its mean, and the lowest possible pressure drops. In this direction, Zerpa et al. [7] studied the geometry effects of the divergent and convergent sections in classical injectors, as did also Almeida [8], but this one with the aim of improving the propulsion of the fluid at the injector outlet. Zhang [9] focused on studying how the dimensional parameters affect the pressure distribution inside the injector, concluding that the throat length does not affect the pressure distribution inside the injector. Also analyzing these variables, in [10], aspect ratios were established that sought the maximum allowable differential pressure for the injector to operate at the maximum flow rate of the injector. However, Park [11] established a maximum differential pressure criterion as a function of Reynolds number.

A typical effect sought in the use of Venturi injectors is the generation of vacuum, but at a certain point the depression can produce cavitation, and given the importance of this effect, Shi et al. and Bermejo et al. [12,13] studied how this cavitation affects and its possible uses. One of these uses is fluid mixing; thus, Shi and Nikrityuk [14,15] studied how this cavitation affects the mixing process.

O’Hern et al. [16] established a method for the design of these injectors, while Sobenko et al. [17] proposed a model for predicting the performance of a Venturi injector, in addition to analyzing the flow pressure ratios that generated the highest level of instability in the Venturi injector.

On the other hand, there are studies focused on powder transport or injection. This use of Venturi injectors has been well established for many years [18], but progress is still being made in understanding the internal phenomena of the injector. When powder is introduced both in virtual tests [19] as well as in real tests [20], it should be noted that the requirements for fluid transport are different from those for powder transport, and so the design of injector geometry continues to be studied and advanced, as did by Xu et al. [21]. Of course, it is necessary to study how the sprayed powder behaves depending on its use [22,23].

In the case of powder transport, it must also be taken into account how particle morphology affects injector performance [24].

In terms of injector optimization, Expósito et al. [25] established a procedure applied to classical injectors focused on fluid mixing by using genetic algorithms, and Wang et al. [26] performed another optimization, also applying it to fluid mixing, but using a somewhat different Venturi injector design and machine learning algorithms as the optimization method.

In all the above studies, no work can be found that focuses on the design of an optimal Venturi with a view to maximizing powder transport as a priority. It is also noted that the powder transport studies focus on Venturi injectors that feed powder by gravity, not by suction.

In addition, if the DED manufacturing process requires an inert gas (normally at high pressure), the powder feed needs to be at lower pressure to guarantee the fast cleaning of the atmosphere. Thus, the transport of powder at atmospheric pressure is a good option, but no studies have been found that focus on the design of low-pressure Venturi injectors (less than 100 kPa) [18].

Based on these detected shortcomings, this study optimized the different dimensional parameters of a Venturi injector to maximize the amount of powder capable of suction and transport for a specific flow in a range of low pressures and with high transport precision for the development of a mixing powder device for Functionally Graded Additive Manufacturing.

## 2. Materials and Methods

**Figure 2:**
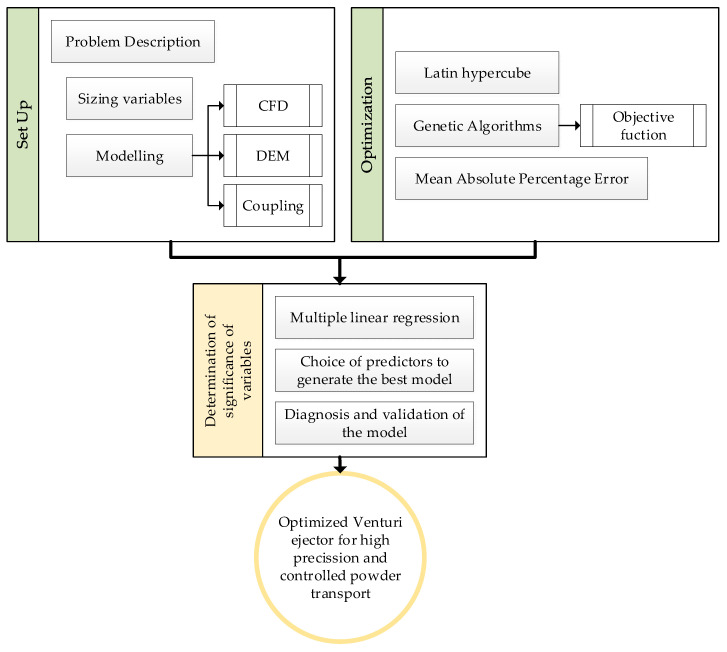
Flowchart of the research process.

This work presents a methodology for optimizing the dimensions of Venturi injectors with low pressure for the transportation of powder with high accuracy. To achieve this, this section presents the description of the problem and the main dimensions of Venturi injectors; then, the modeling of the problem (both from the point of view of the flow, CFD, particle interaction, DEM, and coupling between them); and finally, the optimization tools used to obtain the optimal design. Alongside this, the final section explains the statistical tools used to determine the significance of variables with the results obtained. This process follows the flowchart shown in Figure 2.

### 2.1. Problem Description

The objective of this work is to establish a sizing methodology for optimized Venturi injectors, using particle and flow characteristics as a reference.

The mean particle diameter of the powder to be conveyed and the flow velocity were used as reference constants.

In this case, a powder named Powder-ID256-Scale_1.00 was used, which can be found in the database of representative but not real calibrated powders in Altair Edem software^®^ (2022 version) and has the characteristics presented in Table 1. For the flow rate, a volumetric flow rate of 0.01 $m^{3}/s$ was considered.

**Figure 3:**
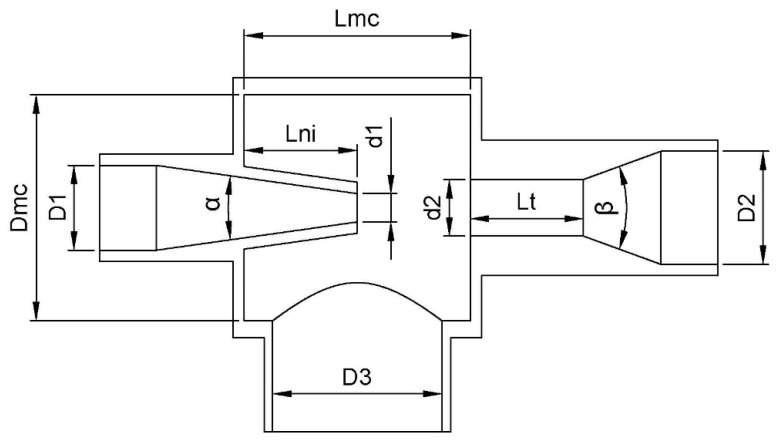
Venturi injector design section and its variables.

Since it is not entirely clear which factors influence powder suction, the dimensional variables shown in Table 2 were taken as the objects of our study. These variables are depicted in Figure 3.

Due to the physical constraints of possible laboratory manufacture, although the variables D1, d1, and D2 have a percentage relationship with other variables, if the result of this relation is greater than 34 mm, this value will be taken as the dimension.

### 2.2. Modeling the Problem

To carry out this study, a simulated environment was developed. For these simulations, 3 interactions must be taken into account: the fluid, the particle–particle interaction, and the interaction between the fluid and the particles. In addition, the simulation interval time must be the minimum possible with the objective of reducing the computational time, but enough to guarantee powder ejection from the Venturi. In this study, that time was 2 s.

#### 2.2.1. Modeling the Fluid Motion

For fluid modeling, the fluid was taken as a continuous element and was governed by the Navier–Stokes continuity equations. In general, applying the conservation of mass to a control volume, the equation in differential form corresponds to Equation (1):

(1) $$\frac{\partial \rho }{\partial t}+\nabla \cdot \left(\rho \vec{u}\right)=0$$

where *ρ* is the density of the fluid; t is the time; and $\vec{u}$ is the flow velocity vector.

In turn, the conservation of motion quantity equation is obtained by applying Newton’s second law to the same control volume, where Equation (2) governs it, and its components can be viscous, pressure, gravity, centrifugal forces, Coriolis forces, etc.:

(2) $$\rho \;\frac{\partial \vec{u}}{\partial t}+\left(\rho \vec{u}\cdot \nabla \right)\vec{u}=-\nabla p+\rho g+\nabla \cdot \tau$$

where p is the pressure; g  is the gravity; and τ is the viscous stress vector corresponding to Equation (3), where the vector $\vec{u}$ is decomposed in the $\vec{i}$, $\vec{j}$, and $\vec{k}$ directions with the following subscript:

(3) $$\tau_{ij}=\mu_{f}\left(\frac{\partial u_{i}}{\partial x_{j}}+\frac{\partial u_{j}}{\partial x_{i}}\right)-\frac{2}{3}\mu \delta_{ij}\frac{\partial u_{k}}{\partial x_{k}}$$

where $\mu_{f}$ is the dynamic viscosity of the fluid.

In addition, the Spalart–Allmaras turbulent flow model [27] was added, which is governed by Equation (4):

(4) $$\frac{\partial \left(\rho \hat{\upsilon }\right)}{\partial t}+\frac{\partial \left(\rho \hat{\upsilon }\bar{u_{j}}\right)}{\partial x_{j}}=\frac{1}{\sigma }\left(\frac{\partial }{\partial x_{j}}\left[\left(\mu_{f}+\rho \hat{\upsilon }\right)\frac{\partial \hat{\upsilon }}{\partial x_{j}}\right]+\rho C_{b2}\frac{\partial \hat{\upsilon }}{\partial x_{j}}\frac{\partial \hat{\upsilon }}{\partial x_{j}}\right)+P+D$$

where σ=2/3 and $C_{b2}=0.622$. Also, P and D are the production and destruction terms of the modified turbulent viscosity, corresponding to Equations (5) and (9), respectively:

(5) $$P=\rho C_{b1}\hat{S}\hat{\upsilon }$$

with

(6) $$\hat{S}=\sqrt{2\Omega_{ij}\Omega_{ij}}+\frac{\hat{\upsilon }}{\kappa^{2}d^{2}}f_{v2}$$

(7) $$\;\Omega_{ij}=\frac{1}{2}\left(\frac{\partial \bar{u_{i}}}{\partial x_{j}}-\frac{\partial \bar{u_{j}}}{\partial x_{i}}\right)$$

(8) $$f_{v2}=1-\frac{x}{1+xF_{v1}}$$

where $\Omega_{ij}$ is the rotation tensor; d is the distance from the nearest wall; κ is the Von Karman’s constant (0.41); and $C_{b1}=0.1355$.

(9) $$D=-\rho C_{w1}F_{w}{\left(\frac{\hat{\upsilon }}{d}\right)}^{2}$$

with

(10) $$F_{w}=g{\left(\frac{1+C_{w3}^{6}}{g^{6}+C_{w3}^{6}}\right)}^{1/6}$$

(11) $$g=r+C_{w2}(r^{6}-r)$$

(12) $$r=\frac{\hat{\upsilon }}{\hat{S}\kappa^{2}d^{2}}$$

Moreover, for the modeling of the turbulent viscosity, Equation (13) was used:

(13) $$m_{t}=\rho \hat{\upsilon }F_{v1}$$

with

(14) $$F_{v1}=\frac{x^{3}}{x^{3}+C_{v1}^{3}}$$

(15) $$x=\frac{\hat{\upsilon }}{\nu }$$

This model was completed with the coefficients $C_{w1}=\frac{C_{b1}}{\kappa^{2}}+\frac{1+C_{b1}}{\sigma^{2}}$, $C_{w2}=0.3$, $C_{w3}=2.0$, and $C_{v1}=7.1$.

#### 2.2.2. Modeling of Particle Interaction

Different mathematical models can be used for particle modeling, depending on the particle characteristics.

**Figure 4:**
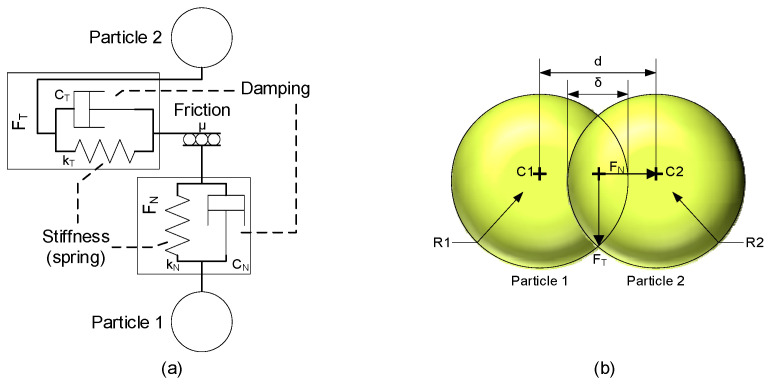
Representation of the interaction between particles (**a**) and of the overlap at the point of contact (**b**).

When talking about polymeric particles, it is assumed that they have a certain level of elasticity. This means that when the particles interact with the walls, they undergo some form of deformation; in the case of particle-to-particle interactions, this deformation causes the distance between their centers to be reduced due to what is considered an overlap between the two particles Figure 4b.

In Figure 4a, these interactions are represented at the point of contact of two particles by a parallel connection between a spring and a damper.

**Figure 5:**
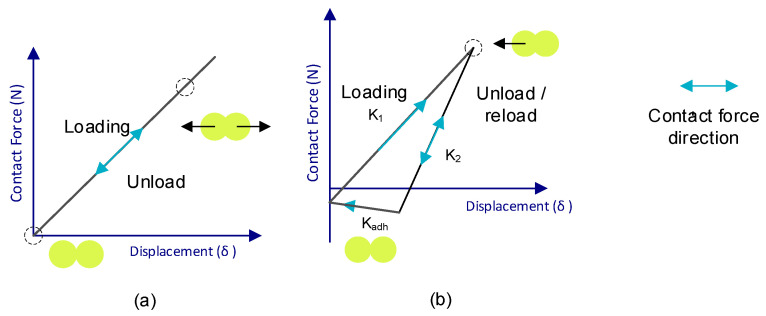
Schematics of the contact–displacement force function of the Hert–Mindlin (**a**) and Edinburgh Elasto-Plastic Adhesion (**b**) models.

Depending on the mathematical model used, the energy transferred by these interactions is represented to a greater or lesser extent in the equations of motion. An example of this is shown in Figure 5, where the contact force–motion functions are depicted: Figure 5a is the representation of the most basic contact model (Hert–Mindlin contact model) [28,29,30,31,32], and Figure 5b depicts the Edinburgh Elasto-Plastic Adhesion contact model.

The equations governing the Hert–Mindlin (no slip) model are summarized in Table 3 [33].

Although each of these models is more complete than the previous one, the complexity of these models results in a high computational cost, so it is not always more effective to choose the most complete model.

In the case of the present work, the Hert–Mindlin (no-slip) model was used for the particle–wall interactions, and the Edinburgh Elasto-Plastic Adhesion model was employed for the interaction between particles.

When two particles come into contact, the force is distributed into two components called the normal force ($F_{N}$) and tangential force ($F_{T}$), as can be seen in Figure 4b. The definition of each of these forces depends on the model.

In the case of the Edinburgh Elasto-Plastic Adhesion model, some factors are added to account for particle plasticity. The governing equations are summarized in Table 4 [34].

#### 2.2.3. The Particle Flow Coupling Model

**Figure 6:**
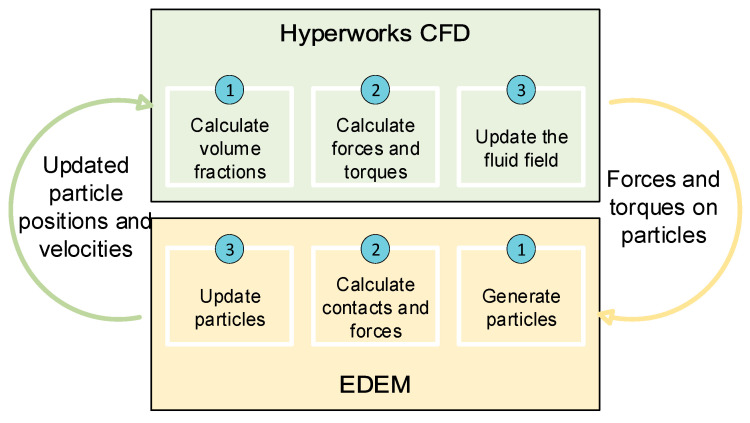
Workflow between Hyperworks CFD and EDEM.

To study the behavior of the particles in the fluid in motion, a joint computational fluid dynamics (CFD) and discrete element method (DEM) simulation was carried out, using the models explained above, in each simulation time step. For this purpose, the software tools Altair Edem^®^ (2022 version) and Altair Hyperworks CFD^®^ (2022 version) were run together, following the workflow shown in Figure 6.

In this way, the workflow starts in the Hyperworks CFD tool, which calculates the volume fractions, forces, and moments of the fluid; it then updates the information for that time step and transfers it to the EDEM tool.

In the EDEM tool, the forces and contacts of the particles are calculated; the position and velocity of the particles are updated; and this information is transferred to the Hyperworks CFD tool.

This cycle was repeated with each time step until the end of the set test time.

To obtain an accurate calculation of the trajectory of these particles, it is necessary to use a mathematical entrainment model that transfers the information between the two phases of the test (fluid and powder). In this study, a model [35,36] that combines the Ergun [37] and Wen-Yu [38] models was used to evaluate the tensile force, $F_{D},$ according to Equation (16):

(16) $$F_{D}=\frac{\beta V_{i}\left|u-v_{i}\right|\left(u-v_{i}\right)}{\left(1-\varepsilon \right)}$$

where

(17) $$\beta =\left\{\begin{array}{cc} \beta_{Ergun}=150\frac{{\left(1-\varepsilon \right)}^{2}µ}{2\varepsilon R}+1.75\left(1-\varepsilon \right)\frac{\rho }{2R}\left|u-v_{i}\right| & \varepsilon <0.8\\ \beta_{Wen-Yu}=\frac{3}{4}C_{D}\rho \varepsilon^{-1.65}\left(1-\varepsilon \right)\left|u-v_{i}\right| & \varepsilon \geq 0.8 \end{array}\right.$$

(18) $$Re=\frac{2R\rho \varepsilon \left|u-v_{i}\right|}{µ}$$

(19) $$C_{D}=\left\{\begin{array}{cc} \frac{24}{Re} & Re\leq 0.5\\ \frac{24}{Re}\left(1+0.15{Re}^{0.687}\right) & 0.5<Re\leq 1000\\ 0.44 & Re>1000 \end{array}\right.$$

where u is the gas velocity vector; $V_{i}$ is the volume of particle I; $v_{i}$ corresponds to the solid velocity vector; and ε is the free volume fraction.

### 2.3. Optimization Using Genetic Algorithms

A process for choosing what variable is modified and the measure of this change is needed. In this study, the process selected is the use of genetic algorithms.

Genetic algorithms are a mathematical assimilation of the law of natural selection in nature [39,40]. In this case, 100 generations with 100 individuals each were applied. A tournament selection was applied by selecting two random individuals and comparing the fitness function value (using the Kriging metamodel interpolation method to estimate the possible responses [41,42]) to store the best one in the intermediate population (100 tournaments until obtaining an intermediate population of 100 individuals). Half of the intermediate population (50% crossover rate) was randomly selected and combined to obtain offspring (arithmetic crossover), while the other half of the intermediate population was maintained. Additionally, a 60% mutation rate was applied, as well as elitism (the worst individual of the new populations was always replaced by the best one of the previous populations). To create these metamodels, the results previously obtained in a design of experiments were used as a reference.

The Kriging metamodel uses an exponential correlation model and a polynomial regression model. However, polynomial regression is able to change the order from 0 to 2, depending on the recorded data. When you have a lower-order polynomial, it requires fewer reference values and is easier to calculate, but it has less precision. For this, the algorithm starts by trying to make use of a second-order regression model to predict each response; if this fails, it will automatically use a first-order regression model, and if this fails, it will go back down using a zero-order regression model. This automatic order change ensures that you are always using the best possible regression model with the data you have.

The modified Latin hypercube algorithm is used to generate a set of initial cases for initial interpolations [39]. First, a combination is generated with all the variables at the minimum of their range, another combination with all variables at the midpoint of their range, and another combination with all the variables at their maximum point, which implies 3 designs. Subsequently, the Latin hypercube algorithm is applied to add “n” points, where “n” is the number of design variables. Therefore, this algorithm divides the range of each variable into equal parts, selects a random value for each variable, and eliminates the corresponding range for each of the variables from the list of possible values to be used in the next combination of variables. This process is repeated until “n” points are added. This means that the optimization will start with a total of 3+n combinations, which in this study means a total of 12 initial combinations.

Since the Kriging metamodel interpolation method gives better results interpolating than extrapolating, the Latin hypercube algorithm was modified so that all generated values corresponding to the first or last rank of each variable are modified and set to the minimum or maximum value, respectively. In this way, these data are shifted to the contour of the search space, promoting data interpolation over extrapolation.

In this study, the Venturi design variables explained in Section 2.1 were used as the variables to be optimized, while the mass of powder discharged in two seconds (Obj^1^) and the pressure drop produced between the inlet and the outlet of the Venturi injector (Obj^2^) are the response variables to maximize and minimize, respectively. To achieve this in a mono-objective approach, the objective function was chosen to transform the scale of the powder discharge and pressure drop results by normalizing them. For this purpose, Equation (20) transforms the minimum value of each variable into 0 and the maximum value into 1, scaling the rest of the results. This transformation is called normalization.

(20) $$x_{norm}=\left(x-\min\left(x\right)\right)/(\max\left(x\right)-\min\left(x\right))$$

Thus, the objective function will be the maximization of Equation (21):

(21) $${Obj}_{normalised}^{1}-{Obj}_{normalised}^{2}$$

The mean absolute percentage error (also known as MAPE) was used as a stopping criterion in each iteration [41], which measures, in percentage terms, the absolute error committed by the response variables as a whole. This indicator responds to Equation (22):

(22) $$M=\frac{1}{n}\sum_{t=1}^{n}\left|\left(A_{t}/F_{t}\right)/A_{t}\right|$$

where n is the number of response variables to optimize; $A_{t}$ is the reference value (those obtained in the simulations to check); and $F_{t}$ is the value to be compared (those given by the algorithm as an estimated value according to the data available for training the Kriging metamodel).

When this criterion is below 4%, the optimization will be considered good since the subsequent numerical simulation of the solution proposed by the genetic algorithm approximately matches (MAPE < 4%) the estimated results of the algorithm. However, if the iteration reaches 4% but better results have been obtained in previous iterations, it continues iterating until the objective function is the best of all cases studied while the maximum MAPE criterion allowed is met. Sometimes the stopping criterion is not reached after many iterations. In these cases, every 10 iterations without reaching the stopping criterion, the results of the MAPE are analyzed. If the trend of these results is towards 4%, 10 more iterations are performed. In case the results start to fluctuate with no improving trend, the best result of the objective function in the last 10 iterations will be assumed as the optimum.

**Figure 7:**
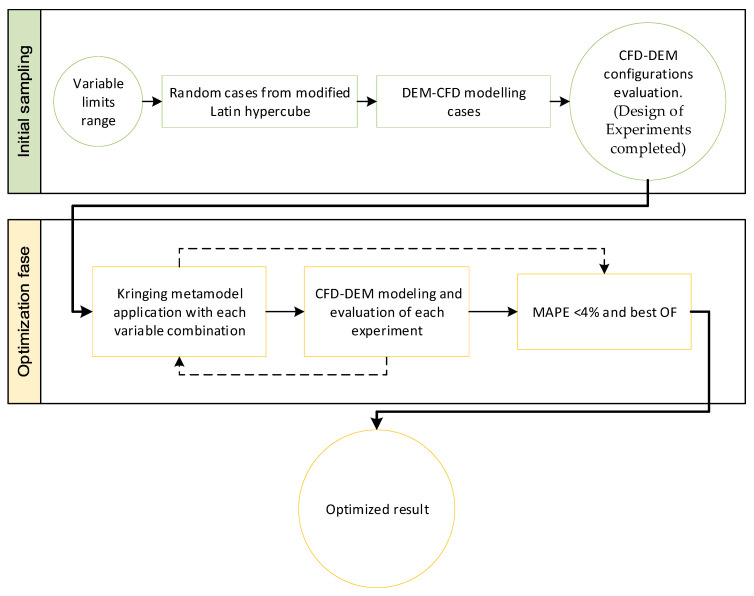
Optimization workflow.

The duty cycle for each iteration can be seen in Figure 7.

### 2.4. Determination of Significance of Variables

In order to study the relative importance, from a statistical point of view, of the different design variables considered, a multiple linear regression analysis with standardized coefficients was carried out to model the behavior of powder discharge.

#### 2.4.1. Multiple Linear Regression

The objective of regression analysis is to mathematically model the behavior of a response variable (Y) as a function of one or more independent or predictor variables ($X_{1},\;X_{2},\;\ldots ,\;X_{k}$).

The multiple linear regression model can be written in matrix notation as follows:

(23) Y=Xβ+ε

where

(24) $$Y=\left[\begin{array}{c} y_{1}\\ y_{2}\\ \vdots \\ y_{n} \end{array}\right],\;X=\left[\begin{array}{ccccc} 1 & x_{11} & x_{21} & \cdots & x_{k1}\\ 1 & x_{12} & x_{22} & \cdots & x_{k2}\\ \vdots & \vdots & \vdots & \cdots & \vdots \\ 1 & x_{1n} & x_{2n} & \cdots & x_{kn} \end{array}\right],\;\beta =\left[\begin{array}{c} \beta_{0}\\ \beta_{1}\\ \vdots \\ \beta_{k} \end{array}\right]\;and\;\varepsilon =\left[\begin{array}{c} \varepsilon_{1}\\ \varepsilon_{2}\\ \vdots \\ \varepsilon_{n} \end{array}\right]$$

The vector β contains the coefficients of the model, where $\beta_{0}$ is the ordinate at the origin and $\beta_{j}$, 1≤j≤k is the average effect that a one-unit increase in the predictor variable $X_{j}$ has on the dependent variable Y, when all the other regressor variables are held fixed or constant. The vector ε contains the errors or residuals, where $\varepsilon_{i}$ is the difference between the observed value and the value estimated by the model. In addition, $\varepsilon_{i}\approx N\left(0,\sigma_{\varepsilon }\right),\;1\leq i\leq n$.

Since the magnitude of each regression coefficient depends on the units in which the predictor variable to which it corresponds is measured, in order to determine the impact of each variable on the model, standardized coefficients were used, which were obtained by standardizing (subtracting the mean and dividing by the standard deviation) the predictor variables prior to the model’s fitting.

Multiple linear correlation models require the following conditions:

- Linearity between the independent variables of the model and the response variable.
- Normality of the residuals: It is assumed that the residuals are normally distributed with a zero mean. This is usually checked by means of graphical methods (histograms, box-and-whisker plots, or quantile–quantile (Q–Q) plots) as well as normality hypothesis tests such as, for example, the Shapiro–Wilk test, which is applicable when analyzing small samples (composed of less than 50 elements) or the Anderson–Darling test, which is a non-parametric test on sample data (with more than 7 elements) coming from a specific distribution.
- Homogeneity of the variance of the residuals (homoscedasticity): To check this, the residuals are plotted. If the variance is constant, they are randomly distributed with the same dispersion and without any specific pattern. One can also resort to homoscedasticity tests such as the Breusch–Pagan test, which only detects linear forms of heteroscedasticity, or the Goldfeld–Quandt test, which compares the variances of two sub-models separated by a specified break point and rejects if the variances differ.
- The residuals are independent of each other: The Durbin–Watson test allows for the diagnosis of the presence of a correlation between consecutive residuals ordered in time, which is a possible manifestation of a lack of independence.
- There are no outliers with a high influence. That is, the regression model is not strongly influenced by one or more outlier data points because this would raise doubts about the adequacy of the model and the reliability of the data in some cases. Cook’s distance allows for detecting outliers with a high influence. For a sample of n elements and a model of k independent variables, a Cook’s distance greater than the median of an F-distribution with p and n−p degrees of freedom, with p=k+1, is considered of concern.
- Uncorrelated predictors: In multiple linear models, the predictors must be independent, meaning there must be no collinearity between them. Tolerance and the variance inflation factor (VIF) are two parameters that quantify the same thing (one is the inverse of the other). The reference limits that are usually used are: VIF=1: absence of collinearity; 1<VIF<5: regression may be affected by some collinearity; 5≤VIF≤10: cause for concern; and VIF>10: serious collinearity.
- Parsimony: This term refers to the fact that the best model is the one that can most accurately explain the variability observed in the response variable using the least number of predictors.

Good models are those that meet the most criteria for goodness-of-fit. There will always be circumstances where failure to meet any of the criteria will not necessarily make the model unfeasible from a practical point of view [42].

#### 2.4.2. Choice of Predictors to Generate the Best Model

When selecting the predictors to be part of the model, several methods can be followed, among them the so-called stepwise methods, which consist of iteratively adding and/or removing predictors in the regression model in order to find the subset of variables in the dataset that results in a model that reduces the prediction error. There are three stepwise regression strategies:

- Forward selection, which starts with no predictors in the model, iteratively adds the most contributing predictors, and stops when the improvement is no longer statistically significant.
- Backward selection (or backward elimination), which starts with all predictors in the model (full model), iteratively eliminates the least contributing predictors, and stops when you have a model in which all predictors are statistically significant.
- Stepwise selection (or sequential substitution), which is a combination of the forward and backward selections. You start with no predictors and sequentially add the most contributing predictors (as in forward selection). After each new variable is added, any variable that no longer provides an improvement in model fit (as in backward selection) is removed.

The step-by-step method requires some mathematical criteria to determine whether the model gets better or worse with each addition or removal. There are several parameters or metrics that can be used, the most important of which are: $R_{adjusted}^{2}$, Mallows’ $C_{p}$, the Akaike Information Criterion (AIC), and the Bayesian Information Criterion (BIC) [43].

When there are terms in the model that do not contribute significantly to the model, $R_{ajusted}^{2}$ tends to be smaller than $R^{2}$. Therefore, it is desirable to refine the model. In general, to speak of a model that has a satisfactory fit, it is necessary that the coefficients $R^{2}$ and $R_{ajusted}^{2}$ have values greater than 0.7. However, when comparing different models for the same dataset, a lower AIC/BIC score is better.

#### 2.4.3. The Lack-of-Fit Test

The F-test is useful for checking that the model as a whole performs better than chance. If a regression model does not produce a significant F-test result, then you probably do not have a very good regression model (or, quite possibly, you do not have very good data). However, while failing this test is a fairly strong indicator that the model has problems, passing the test (i.e., rejecting the null hypothesis) does not imply that the model is good.

Most computer programs specialized in statistics include procedures to perform both simple and multiple regression analyses and usually include variable selection techniques. In this work, we used the free software Jamovi [44] based on the statistical language R [45,46].

## 3. Results

### 3.1. Optimization of Variables

**Figure 8:**
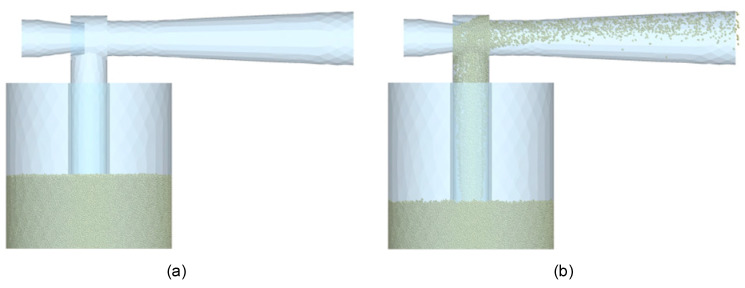
TSV2 simulation starting (**a**) and ending (**b**).

Given the limits for each variable explained in Section 2.1 and applying the modified Latin hypercube algorithm, a total of 12 initial cases were obtained, which can be seen in Table 5 (design variables). One example of the simulation is depicted in Figure 8, which corresponds to the beginning (Figure 8a) and the end (Figure 8b) of the TSV2 simulation.

**Figure 9:**
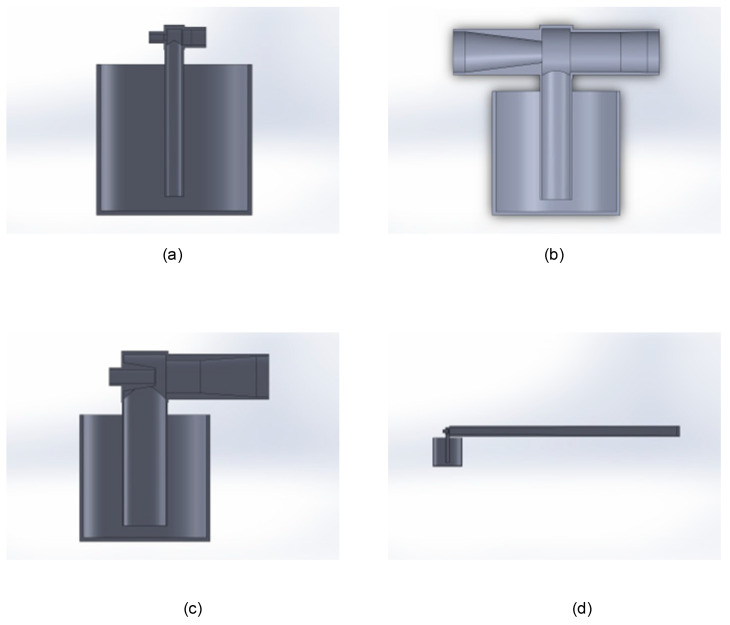
Examples of CAD designs of different configurations: TSV1 (**a**), TSV5 (**b**), TSV7 (**c**), and TSV11 (**d**).

Each of these cases was drawn in CAD, and joint CFD-DEM tests were carried out. These gave the results shown in Table 6. As a reference, some examples of these design configurations are shown in Figure 9.

The direct measurements from the tests can be seen in columns 2 and 3, along with the normalized values in columns 4 and 5.

These data were fed into the optimization algorithm, as explained in Section 2.3, following the cycle of optimization iterations up to a total of 13 times.

For each combination, the genetic algorithm shows, as a result, a possible optimal variable combination and the predicted objective function result. Those optimal design variables obtained by the algorithm after each execution are shown in Table 7.

Table 8 shows the simulation results (both measured and normalized) obtained for every combination in Table 7 (optimal designs). It can be seen how the value of the objective function, given by Equation (21), increases as the optimal point is approached. A deeper analysis of these results is made in Section 3.2.

With each iteration, the predictions of the algorithm results were noted down to apply the stopping criterion. Table 9 shows, for each optimal design, the normalized response variables (powder discharge and pressure drop) according to the simulations (first column) and the same response variables predicted by the genetic algorithm (second column). The MAPE between the estimated and simulated results is depicted in the last sub-column (M). As the number of data points increases, the estimates of the genetic algorithm are more accurate, consequently reducing the MAPE (compared to the simulation results).

After a total of 13 iterations, the results of the proposed optimization algorithm can be considered good since the results estimated by it and those resulting from evaluating this combination of variables through the simulations are less than 4% apart, fulfilling the condition of having the best result of the objective function up to that iteration. It is noteworthy that the optimal result discharges 85% more powder (Obj 1_normalized_) with only a 2% pressure drop (Obj 2_normalized_) compared to all cases studied during the optimization process.

In this iteration 13, the variables obtained were those depicted in Table 10.

**Figure 10:**
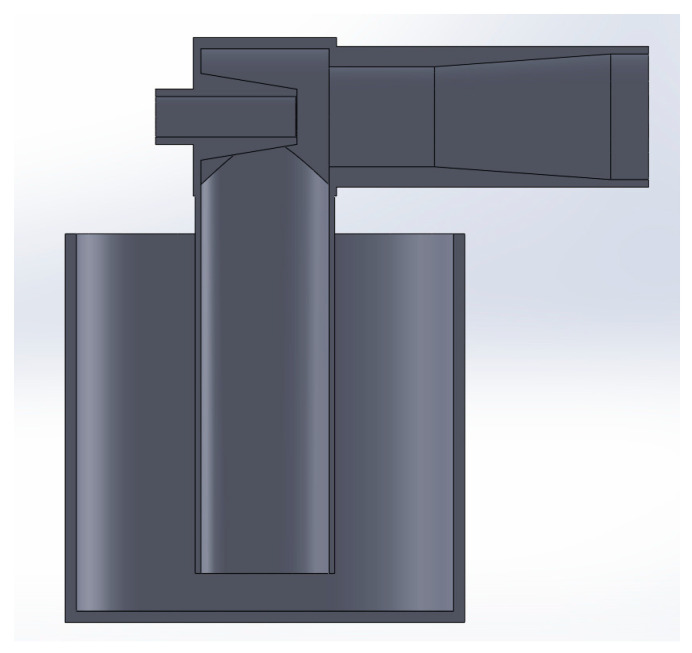
Optimal Venturi (representation in section view).

A CAD representation of the optimal Venturi proportions is shown in Figure 10.

It is worth mentioning that the dimensions of Table 10 are the optimized ones for a 1 mm particle diameter, but they have a proportional relationship with the particle diameter. Therefore, the optimized dimensions could be explained as follows: D3 and d2 are 34 and 26.5 times the particle diameter, respectively; d1 is 40% of d2; D1 is 101% of d1; D2 is 126% of d2; and PRi is 75% of D3. The Lt, α, and β are fixed. Nevertheless, the statistical analysis depicted in Section 3.2 reveals that the most significant design variables of a Venturi injector to maximize the powder discharge are D3, d1, and d2.

**Figure 11:**
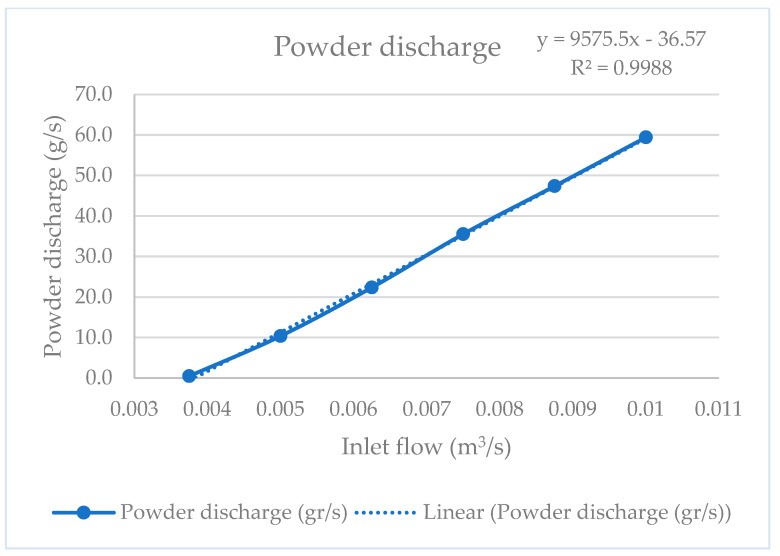
Discharge curve.

**Figure 12:**
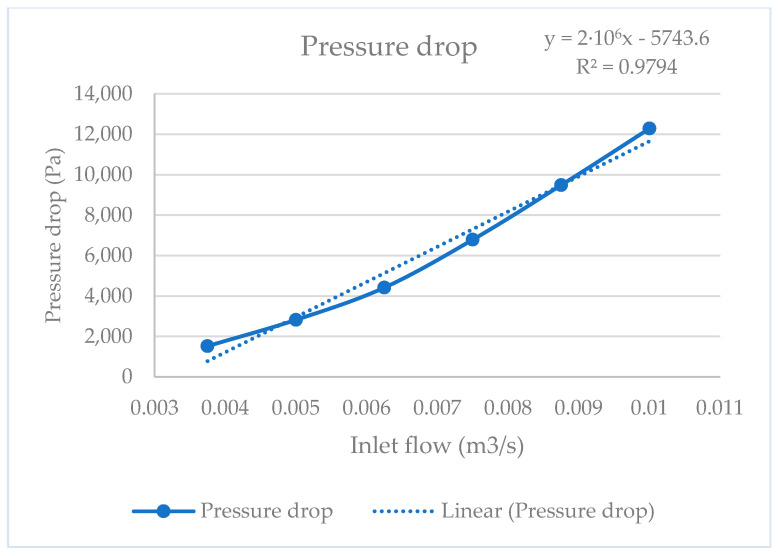
Pressure drop curve.

On the other hand, since the final goal of the optimization of the Venturi injector is to obtain an accurate powder transport system for directed energy deposition, the optimal Venturi injector with a 40 mm D1 and D2 configuration was analyzed using different inlet flow values. This 40 mm D1 and D2 configuration was selected to facilitate standardization and future experimental implementation. Figure 11 and Figure 12 show the results obtained, where a linear relationship between powder discharge and the pressure drop with inlet flow can be observed, respectively.

### 3.2. Determination of the Significance of Variables

A statistical analysis of the powder discharge results is now carried out in order to find out to what extent each of the variables used in the design of the Venturi injector affects the results.

**Figure 13:**
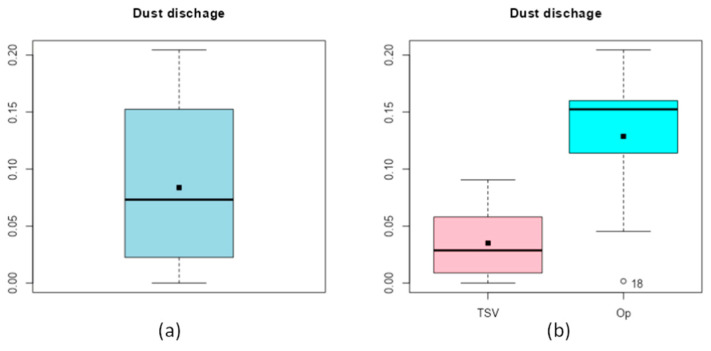
Grouping of the results all together (**a**) and separated by origin (**b**).

The first thing to carry out is to group all the results together to see the dispersion of the results. Figure 13a shows that the results are not very dispersed. However, when the results of the randomly generated variations (TSV) are separated from the results of the optimizations (Op), it is clear that the optimizations tend to be upward (Figure 13b).

#### 3.2.1. Multiple Linear Regression

For the construction of the models, a backward selection based on the *p*-value was first used to establish an order of inclusion of the variables by blocks (one by one) in the Model Builder of the Linear Regression library of the jmv Module in Jamovi software (2.3.28 version) [46].

Bearing in mind that it is of interest to have $R^{2}$ and $R_{adjusted}^{2}$ coefficients greater than 0.7 and low AIC and BIC values, with a *p*-value for the overall model test as small as possible, it was decided to choose Model 5 to study the relative importance, from a statistical point of view, of the different design variables considered as it fulfills more criteria of the quality of fit (see Table 11).

In Model 5, the most significant variables are D3, d1, and d2 (in that order), all of them with a *p*-value of less than 0.05 (see Table 12). Furthermore, the values of the standard estimators and the corresponding 95% confidence interval show that, if the other variables of the model are kept constant, the higher the value of D3, the higher the powder discharge increases, and the higher the values of d1 and d2, the powder discharge decreases in both cases.

Regarding the Cook’s distance (see Table 13), as the number of sample data is n=25 and the number of independent variables is k=5 (p=k+1), we obtain **qf**(0.50, p, n-p) = 0.9238, where **qf**(p, df1, df2) is a quantile function for the F distribution with df1 and df2 degrees of freedom of the R Stats Package [45].

Consequently, as the maximum Cook’s distance does not exceed this value, no outliers with high influence are detected, as can be seen in Figure 12.

#### 3.2.2. Checking Assumptions

The verification tests explained in Section 2.4 were carried out, and the results that were obtained are shown in Table 14 and Table 15.

Since in all the tests the *p*-value is greater than 0.05, there is no reason to assume that the assumptions of normality, homogeneity of variance, and independence of residuals are violated.

**Figure 14:**
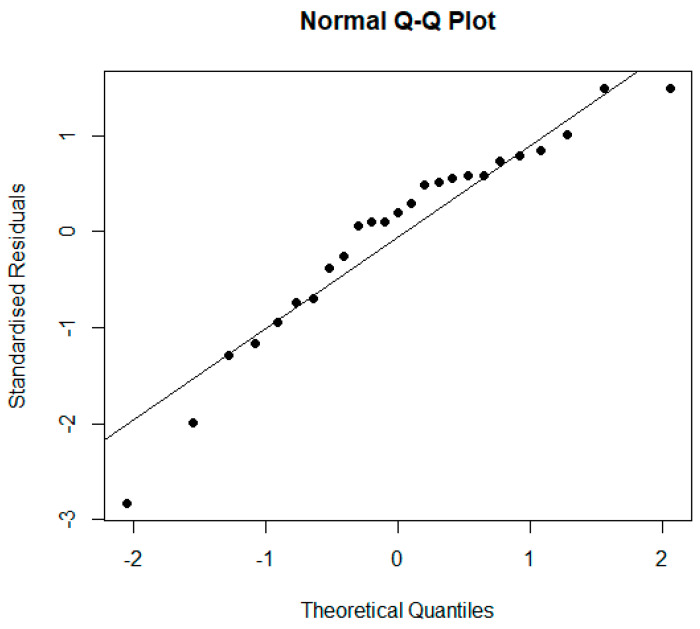
Q-Q plot.

In the Q-Q plot (Figure 14), the points are quite close to the line, thus corroborating the normality of the residuals.

Finally, a collinearity study of the variables was carried out, the results of which can be seen in Table 16.

In this case, as the VIF values are closer to one than to five, it can be concluded that although the regression may be affected by some level of collinearity, this is not a cause for concern, as indicated in Section 2.4.1.

## 4. Conclusions

After evaluating the different combinations of the dimensional variables required for the manufacture of low-velocity Venturi injectors using combined CFD and DEM simulations, a genetic algorithm was used to achieve an optimal combination of variables for sizing a Venturi injector based on mean particle diameter and flow rate.

From analyzing these results, it can be seen that, by aiming for the lowest possible pressure drop, the Venturi convergence and divergence angles (α and β) are within the ranges of the standard for the manufacture of classic Venturi injectors. Therefore, they can be considered valid.

Furthermore, from the statistical study, it can be concluded that the variables that most significantly affect powder suction are D3, d1, and d2 (in that order), which makes it clear that d1 and d2 are important because they are the two variables on whose size the vacuum generated in the mixing chamber depends, and at equal pressure, the larger the suction diameter (D3), the more physical space there is for the particles to rise.

This suggests that the switching variables D1 and D2 can act independently, allowing them to be adapted to the system where the injector is to be installed.

It is also confirmed that throat length does not affect powder deposition [9], thus making it possible to reduce the size of the injector. Additionally, the position of the nozzle inside the mixing chamber is similar to that demonstrated by Xu et al. [21].

On the other hand, the simulations of the optimal Venturi configuration with different inlet flow demonstrated a good linearity between inlet flow and powder discharge, which means that it is possible to control the mass flow and, consequently, the powder discharge. With this approach, different Venturi injectors could be integrated for the development of accurate mixing powder devices intended for Functionally Graded Additive Manufacturing. However, this linearity was only demonstrated for flow rates lower than the one used for optimization (0.01 m^3^⁄s), so the optimal parameters should be recalculated for higher flow rates. Since the objective of this study is the use of this optimized injector for a DED system, the recommendation is to optimize the injector for the maximum flow rate required in each case. In this optimization process, we found better ranges of pressure drop than observed by Zerpa et al. [7], and better linearity than observed by Almeida et al. [8] about the press drop–flow relationship. We also demonstrated that, in the case of powder transportation, the relation between the nozzle and throat diameters is above the found in the bibliography. Comparing our results with those obtained by Wang et al. [26] using machine learning, we can see how, while the input and outlet angles are similar, the proportional dimensions of the nozzle and the throat are different, confirming that the optimization is not the same when the feed process is by gravity or suction.

It should be noted that the mathematical models used in this work are fully validated according to the literature. However, future research will be carried out to experimentally validate these results. On the other hand, this optimization procedure is based on the particle diameter, so, in future research, it remains to be seen its effectiveness when applied to particles with different properties such as density, cohesivity, or diameters, even opening the possibility to study a range of optimal dimensions for more than one powder in the same injector.

Having demonstrated that it is possible to use Venturi injectors to unify the metering and conveyance in DEM systems, it has the following advantages over traditional systems: being a unified system whose implementation should be easier and cheaper; easy installation of more nozzles, thus increasing the possibilities of Functionally Graded Materials; and easy change of materials (cleaning and feeding). In view of the results obtained, the use of Venturi injectors could be studied in other technologies such as material extrusion or powder bed fusion, changing the feeding method, and giving the possibility to work with Functionally Graded Materials in different ways.

## Figures and Tables

**Table 1:** Particle data.

**Particle properties**	
Average diameter	1 mm
Standard deviation of diameter	0.15 mm
Poisson’s ratio (ν)	0.25
Solid density (ρ)	490 kg/m^3^
Shear modulus (G)	1.00 × 10^7^ Pa
**Particle–particle interactions**	
Coefficient of restitution	0.3
Coefficient of static friction	0.1
Coefficient of rolling friction	1
**Particle–wall interactions**	
Coefficient of restitution	0.1
Coefficient of static friction	0.5
Coefficient of rolling friction	0.1

**Table 2:** Venturi injector design variables and their constraints.

Variable	Range of Action
Venturi inlet diameter (D1)	101–200% of d1 or 34 mm
Mixing chamber inlet nozzle diameter (d1)	40–100% of d2 or 34 mm
Venturi outlet diameter (D2)	101–200% of d2 or 34 mm
Mixing chamber outlet diameter (d2)	10–33 times the particle diameter in mm
Suction pipe diameter (D3)	10–34 times the particle diameter in mm
Percentage of the length of the mixing chamber where the nozzle is introduced (PRi)	1–75% of the length of the mixing chamber
Venturi throat length (Lt)	1–50 mm
Input convergence angle (α)	1–30 degrees
Exit divergence angle (β)	1–10 degrees

Mixing chamber diameter (Dmc), dependent on and limited by the largest of the variables D1, D2, and D3 in each case (not the design variable). Mixing chamber length (Lmc), dependent on and limited by the greater of the variable D3 in each case (not the design variable). Length corresponding to Lmc multiplied by PRi (Lni).

**Table 3:** Hert–Mindlin equation (no-slip) model.

Normal force	$F_{N}=-\frac{4}{3}E_{eq}\sqrt{R_{eq}-\delta_{n}^{3/2}}$
Tangential force	$F_{T}=-min\left\{S_{T},\delta_{T},{µ}_{s}\left|F_{N}\right|\right\}\cdot t$
Normal and tangential damping force	$F_{N,T}^{d}=-2\sqrt{\frac{5}{6}}\frac{\ln e}{\sqrt{\ln^{2}e\;+\;\pi^{2}}}\sqrt{K_{N,T}m_{eq}}v_{N,T}^{rel}$
Normal and tangential stiffness	$K_{N}=2E_{eq}\sqrt{R_{eq}\delta_{N}}\;;\;K_{T}=8G_{eq}\sqrt{R_{eq}\delta_{N}}$
Equivalent Young’s modulus	$\frac{1}{E_{eq}}=\frac{\left(1\;-\;v_{i}^{2}\right)}{E_{i}}+\frac{\left(1\;-\;v_{j}^{2}\right)}{E_{j}}$
Shear modulus	$G_{eq}={\left[\frac{2\left(2\;-\;v_{i}\right)\left(1\;+\;v_{i}\right)}{E_{i}}+\frac{2\left(2\;-\;v_{j}\right)\left(1\;+\;v_{j}\right)}{E_{j}}\right]}^{-1}$
Equivalent radius	$\frac{1}{R_{eq}}=\frac{1}{R_{i}}+\frac{1}{R_{j}}$
Equivalent mass	$m_{eq}={\left(\frac{1}{m_{i}}+\frac{1}{m_{j}}\right)}^{-1}$
Rolling friction	$\tau =-{µ}_{r}F_{N}R$

The subscripts *N* and *T* stand for normal and tangential, respectively; i and j correspond to each of the interacting particles; e is the coefficient of restitution; ${µ}_{s}$ represents the coefficient of sliding friction; ${µ}_{r}$ is the rolling coefficient; and v denotes the relative velocity of the corresponding particle.

**Table 4:** **The** Edinburgh Elasto-Plastic Adhesion model.

Normal and tangential force	$F_{N}=\left(f_{hys}+F_{N}^{d}\right)u\;;\;F_{T}=f_{ts}+F_{Td}$
Normal overlap contact force	$f_{hys}=\left\{\begin{array}{cc} f_{0}+K_{1}\delta^{n} & si\;K_{2}\left(\delta^{n}-\delta_{p}^{n}\right)\geq K_{1}\delta^{n}\;\\ f_{0}+K_{2}\left(\delta^{n}-\delta_{p}^{n}\right) & K_{1}\delta^{n}>K_{2}\left(\delta^{n}-\delta_{p}^{n}\right)>-k_{adh}^{x}\\ f_{0}-k_{adh}^{x} & si\;k_{adh}^{x}>K_{2}\left(\delta^{n}-\delta_{p}^{n}\right) \end{array}\right.$
Normal and tangential damping force	$f_{N,T}^{d}=\left\{\begin{array}{cc} \beta_{L}v_{N,T}^{\underset{rel}{\to }} & si\;n=1\\ -2\sqrt{5/6}\beta_{NL}\sqrt{K_{N,T}mv_{N,T}^{\underset{rel}{\to }}} & si\;n>1 \end{array}\right.$
Linear and non-linear damping coefficients	βL=4meqK11 + πln⁡e2 ; βNL=ln⁡eln⁡e2 + π2
Tangential spring force	$f_{ts}=f_{ts(n-1)}+∆f_{ts}$
Increase of tangential spring force	$∆f_{ts}=k_{T}\delta_{T}$
Tangential stiffness	$k_{T}=\zeta_{tm}\left\{\begin{array}{cc} k_{1} & si\;n=1\\ 8G_{eq}\sqrt{R_{eq}\delta_{N}} & si\;n>1 \end{array}\right.$
Rolling friction	$\tau_{i}=-{µ}_{r}f_{hys}R_{i}\varpi_{i}$

u is the unit vector at the center of the particle; $\zeta_{tm}$ represents the stiffness factor; and $\varpi_{i}$ denotes the angular velocity at the point of contact.

**Table 5:** List of initial cases.

Venturi No.	D3 (mm)	d2 (mm)	d1 (%)	D1 (%)	D2 (%)	Lt (mm)	PRi (%)	α (°)	β (°)
TSV1	10	10	40	101	101	1	1	1	1
TSV2	22	21.5	70	150.5	150.5	25.5	38	15.5	5.5
TSV3	34	33	100	200	200	50	75	30	10
TSV4	23.3	18.9	67.4	101.0	175.8	29.3	19.8	30.0	3.5
TSV5	23.5	33.0	53.9	200.0	155.7	41.4	1.0	14.5	2.6
TSV6	13.4	10.0	88.0	183.6	136.8	38.1	30.0	1.0	5.2
TSV7	16.7	13.5	52.7	147.4	117.5	10.6	58.1	7.0	6.9
TSV8	29.8	15.9	84.9	126.1	161.7	18.0	36.5	11.8	7.9
TSV9	34.0	26.9	40.0	135.1	124.0	28.1	75.0	18.7	8.8
TSV10	27.4	29.5	62.1	113.2	101.0	14.7	49.7	7.5	1.0
TSV11	10.0	22.8	79.1	163.3	200.0	50.0	13.2	21.5	4.2
TSV12	19.2	22.7	100.0	170.3	179.5	1.0	66.0	24.2	10.0

**Table 6:** Results of cases generated by the modified Latin hypercube.

	Measured		Normalized	
Venturi No.	Powder Discharge (kg)	Δpi-o (Pa)	Powder Discharge	Δpi-o
TSV1	0.0481	112,787	0.53240	1.00000
TSV2	0.0487	2332	0.53864	0.02183
TSV3	0.0000	47	0.00000	0.00160
TSV4	0.0672	−133	0.74264	0.00000
TSV5	0.0349	1548	0.38593	0.01489
TSV6	0.0131	46,164	0.14454	0.41000
TSV7	0.0780	56,104	0.86294	0.49803
TSV8	0.0092	2359	0.10196	0.02207
TSV9	0.0904	7017	1.00000	0.06332
TSV10	0.0225	203	0.24879	0.00298
TSV11	0.0086	1064	0.09498	0.01060
TSV12	0.0000	377	0.00000	0.00452

**Table 7:** Combinations of variables in the optimization process.

Venturi No.	D3 (mm)	d2 (mm)	d1(%)	D1 (%)	D2 (%)	Lt (mm)	PRi (%)	α (°)	β (°)
Op1	34.00	10.00	40	200	200	50.0	75.00	30.00	1.00
Op2	34.00	10.00	40	200	101	50.0	54.83	30.00	10.00
Op3	10.05	10.96	40	101	200	50.0	75.00	30.00	9.12
Op4	34.00	19.67	40	107	200	1.0	75.00	11.02	5.19
Op5	34.00	10.00	40	101	200	1.0	75.00	1.00	7.25
Op6	34.00	33.00	40	106	200	1.0	75.00	1.00	10.00
Op7	10.35	19.62	40	101	200	1.0	75.00	30.00	1.00
Op8	34.00	26.33	40	101	126	28.2	75.00	18.67	8.28
Op9	34.00	26.39	40	101	125	28.2	75.00	18.69	8.33
Op10	34.00	26.48	40	101	125	28.2	75.00	18.72	8.28
Op11	34.00	26.42	40	101	125	28.2	75.00	18.83	8.27
Op12	34.00	26.50	40	101	126	28.0	74.99	18.72	8.29
Op13	34.00	26.50	40	101	126	28.0	75.00	18.73	8.28

**Table 8:** Results of the optimization process.

	Measured		Normalized		
	Powder Discharge (kg)	Δpi-o (Pa)	Powder Discharge $\left({Obj}_{normalized}^{1}\right)$	Δpi-o $\left({Obj}_{normalized}^{2}\right)$	Objective Function $\left({Obj}_{normalized}^{1}-{Obj}_{normalized}^{2}\right)$
Op1	0.1534	570,583	1.70	5.05	−3.36
Op2	0.1141	507,955	1.26	4.50	−3.24
Op3	0.0732	96,261	0.81	0.85	−0.04
Op4	0.1353	9069	1.50	0.08	1.41
Op5	0.2044	634,533	2.26	5.62	−3.36
Op6	0.0018	2150	0.02	0.02	0.00
Op7	0.0454	6284	0.50	0.06	0.45
Op8	0.1524	2716	1.69	0.03	1.66
Op9	0.1510	1855	1.67	0.02	1.65
Op10	0.1600	2398	1.77	0.02	1.75
Op11	0.1533	2898	1.70	0.03	1.67
Op12	0.1616	2260	1.79	0.02	1.77
Op13	0.1674	2179	1.85	0.02	1.83

**Table 9:** MAPE stop criterion results.

	Normalized (Simulated)		Normalized Estimates by the GA		
	Powder Discharge $\left({Obj\;1}_{normalized}\right)$	Δpi-o $\left({Obj\;2}_{normalized}\right)$	Powder Discharge $\left({Obj\;1}_{normalized}\right)$	Δpi-o $\left({Obj\;2}_{normalized}\right)$	M (%)
Op1	1.70	5.05	3.46	0.74436	94.5
Op2	1.26	4.50	2.38	−0.00001	94.4
Op3	0.81	0.85	1.50	0.00048	92.5
Op4	1.50	0.08	1.08	0.00005	63.9
Op5	2.26	5.62	1.48	0.00000	67.2
Op6	0.02	0.02	1.40	0.00000	3598.6
Op7	0.50	0.06	1.09	0.00000	108.7
Op8	1.69	0.03	0.83	0.00032	74.8
Op9	1.67	0.02	1.65	0.00182	45.4
Op10	1.77	0.02	1.66	-0.00019	53.5
Op11	1.70	0.03	1.73	0.02784	2.9
Op12	1.79	0.02	1.74	0.01459	17.0
Op13	1.85	0.02	1.78	0.02007	2.9

**Table 10:** Final optimized dimensions result.

D3 (mm)	d2 (mm)	d1 (%)	D1 (%)	D2 (%)	Lt (mm)	PRi (%)	α (°)	β (°)
34.00	26.50	40	101	126	28.0	75.00	18.73	8.28

**Table 11:** Statistical results for each regression model.

Measures of Model Fit										
							Global Model Test			
Model	R	R^2^	R^2^ Adjusted	AIC	BIC	RMSE	F	gl1	gl2	*p*-Value
1	0.7112	0.5059	0.4844	−77.87	−74.21	0.04522	23.547	1	23	<0.001
2	0.8006	0.641	0.6084	−83.86	−78.98	0.03854	19.643	2	22	<0.001
3	0.8443	0.7128	0.6718	−87.44	−81.34	0.03447	17.376	3	21	<0.001
4	0.8527	0.7271	0.6725	−86.71	−79.39	0.0336	13.32	4	20	<0.001
5	0.8694	0.7558	0.6916	−87.49	−78.96	0.03178	11.763	5	19	<0.001
6	0.8736	0.7631	0.6842	−86.25	−76.5	0.03131	9.665	6	18	<0.001
7	0.8759	0.7672	0.6713	−84.68	−73.71	0.03104	8.003	7	17	<0.001
8	0.8788	0.7723	0.6585	−83.24	−71.05	0.03069	6.784	8	16	<0.001
9	0.8793	0.7732	0.6372	−81.34	−67.93	0.03063	5.683	9	15	0.002

**Table 12:** Results of standard estimators and confidence intervals at 95%.

Model 5							
						95% Confidence Interval	
Predictor	Estimator	EE	t	*p*-Value	Standard Estimator	Inferior	Top
Constant	0.158285	0.039635	3.994	0.00078			
d1	−0.001301	4.64 × 10^−4^	−2.806	0.01128	−0.413	−0.72107	−0.10494
D3	0.00373	9.22 × 10^−4^	4.044	0.00069	0.5413	0.26117	0.82149
d2	−0.002835	0.001071	−2.647	0.0159	−0.3434	−0.61495	−0.07187
D1	−4.61 × 10^−4^	2.72 × 10^−4^	−1.694	0.16617	−0.2735	−0.61142	0.06444
Lt	7.61 × 10^−4^	5.09 × 10^−4^	1.496	0.15109	0.2046	−0.08166	0.49083

**Table 13:** Cook’s distance of Model 5.

Cook’s Distance				
			Tour	
Average	Median	DE	Min	Max
0.0694	0.01458	0.1152	3.08 × 10^−4^	0.4368

**Table 14:** Normality and heteroscedasticity tests.

		Statistic	*p*-Value
Normality tests	Shapiro–Wilk	0.9304	0.089
	Anderson–Darling	0.5628	0.13
Heteroscedasticity tests	Breusch–Pagan	5.333	0.377
	Goldfeld–Quandt	2.998	0.101
	Harrison–McCabe	0.3547	0.174

Note. Additional results provided by more tests [44].

**Table 15:** Autocorrelation test.

Durbin–Watson Autocorrelation Test		
Autocorrelation	DW Statistic	*p*-Value
0.03302	1.868	0.594

**Table 16:** Study of the collinearity of the variables.

Collinearity Statistics		
	VIF	Tolerance
d1	1.686	0.5932
D3	1.394	0.7172
d2	1.310	0.7635
D1	2.028	0.4930
Lt	1.455	0.6871

## Data Availability

The data presented in this study are available within this article.
